# Lectures by James Syme, Esq.

**Published:** 1855-04

**Authors:** 


### LECTURES BY JAMES SYME, ESQ.

Lecture i. Sinuses of the Hip depending upon Exfoliations from the Pelvis.—If the treatment of diseases be interesting in proportion to the degree in which they affect the patient's comfort and admit of beneficial interference, the case which we have now to consider seems deserving of attention. That the morbid derangement concerned is no trifling matter will appear sufficiently from the fact that the patient has come nearly four thousand miles in quest of relief; and unless the results shall differ from those hitherto experienced under similar circumstances, complete recovery will be speedily accomplished, without any pain or other bad consequences of the means employed.

It is here necessary that you should recollect the distinction between necrosis and caries. In the former disease a portion of bone dies, and separates from the living substance, so that no obstacle to recovery exists after the exfoliation or detached piece escapes from the position where it is situated; but in the latter, the bone retains its vitality, and obstinately remains in a diseased condition, without any natural limit of duration, except the life of the patient or conversion of the caries into necrosis.

It must further be recollected that the dense osseous substance which composes the shafts of bones is chiefly liable to necrosis, and that the spongy or cancellated texture is almost exclusively the seat of caries. Now, sinuses about the pelvis are unhappily met with very frequently as the attendants or consequences of disease in the hip joint, vertebræ, or sacrum, where the disease, being of an incurable kind, and the part concerned not admitting of removal, any sort of treatment can produce no better effect than a very imperfect degree of palliation, and it has hence been usual to regard such cases as of a very hopeless character. But nearly thirty years ago there happened to fall under my notice a case which showed that such a judgment should not be passed as a matter of course, or without more caution and discrimination than had been supposed requisite. The patient was a young man, aged twenty-eight, who for the preceding seven years, had suffered from sinuses about the hip and upper part of the thigh, which being regarded as proceeding from fistula in ano, had been so treated by the late Mr. George Bell and other surgeons, without obtaining the relief desired. He had then applied to quacks with no better success, and finally, abandoning all hope of recovery, had allowed the disease to pursue its course. It was a considerable time after this resolution that my assistance was asked. I found him extremely emaciated, and so weak that he hardly could leave his bed, with a large abscess of the thigh, and several sinuses about the hip, discharging matter profusely. Having opened the abscess, I examined the sinuses, and found that one, which opened in the fold between the buttock and thigh, led to the tuberosity of the ischium, in which there was a cavity containing an exfoliation of bone. I therefore dilated this sinus by incision, introduced my finger, and having found an opening between the origins of the extensor muscles, enlarged it by the bistoury, so as to obtain access to the interior, whence I removed a small bit of dead bone. The patient then quickly recovered, and ever afterwards enjoyed good health.

As there could be no doubt that the exfoliation had caused all the distressing symptoms of this severe and long-protracted case, it became very desirable to ascertain the origin of the evil, which can hardly be attributed to the ordinary influence of cold or external injury. Upon inquiry, it appeared that the patient had first experienced uneasiness after a day's employment in curing herrings, and that the discharge of this duty had required him to stand with his feet apart, alternately stooping and stretching his arms upwards to the full extent, subsequently to which he had felt a painful sense of fatigue in the back part of his thighs. It then occurred to me that the disease had originated from over-exertion of the muscles which arise from the tuberosity of the ischium, and that the bone with which they are there connected had suffered in consequence, so as to exfoliate, instead of being excited to præternatural growth, as Sir Astley Cooper, with apparently good reason, thought likely to happen, from inordinate muscular contraction. Several cases of a similar kind, which afterwards occurred, tended to confirm this suspicion; and in a paper on the subject (*Edinburgh Medical and Surgical Journal* for 1828) I thus expressed my sentiments in regard to the pathological explanation:—"The history of these cases will, I hope, effect the great object of this paper, which is to excite a more discriminating diasnosis and active treatment of sinuses of the pelvis. As to the origin of the exfoliations, I will not at present say much. It seems very evident that they cannot result from the direct effects of violence, since, in all cases detailed, the bone concerned was securely protected by its situation from any such injury. In all of them (if we except the first, where no information could be obtained as to the origin of the complaint) there was violent muscular contraction, and I am inclined to think that this may have been the exciting cause of inflammation and death of the bone.

The subject is curious, and worthy of investigation, but of little importance when compared with the practical benefit which may result from a knowledge of the fact that sinuses of the pelvis sometimes depend upon loose exfoliations, which will not find their way out unassisted, but which may be readily removed artificially with the effect of a speedy and permanent recovery.

We may now proceed to the case which has led to these remarks. The patient is T. C——, aged twenty four, a blacksmith in Toronto, Upper Canada. About a year and a half ago he was trying how far he could leap, when his right leg slipped as he came to the ground, and went out sideways from him. He felt shaken at the time, but did not experience actual pain until next morning, when the right thigh was so painful at its upper part that he could scarcely move; the pain was deeply seated, apparently in the very middle of the substance of the thigh, while the upper part of the abductor muscles was very tender to the touch. The pain continued for about two months, at first preventing him from working, and greatly crippling him, even to the end of this time. A lump then began to form at the extreme upper and inner part of the thigh, a little anterior to the tuberosity of the ischium, and the pain gradually subsided. This swelling remained in nearly the same state for twelve months, when, having attained the size of a hen's egg, it was opened by a surgeon, and discharged about a teacupful of matter. Three weeks afterwards another abscess of larger size formed nearer to the groin, and was followed by several others of smaller extent, which were poulticed, and allowed to open spontaneously, while he lay in the hospital of Toronto, with apparently little prospect of recovery. Two medical gentlemen who had been educated in Edinburgh then became acquainted with the case, and having recognized its similarity to those pointed out by me as dependent upon exfoliation of the pelvic bones, advised the patient to seek my assistance. He accordingly travelled to New York, crossed the Atlantic, and was admitted to this hospital a week ago (on the 30th of October). The patient is now before you. You observe that he is a well-formed, healthy-looking man, and that there is no curvature of the spine, or any other sign of disease, either there or in the hip-joint. There are several sinuses, one in the groin above Foupart's ligament, and others at the junction of the thigh with the perinæum. Through one of the latter the probe passes towards the ascending ramus of the ischium, and there detects a loose piece of bone, which we shall now endeavor to remove.

[Chloroform having been administered, Mr. Syme dilated the sinus by incision, introduced his finger up to the origin of the abductor muscles, and having there felt a small aperture, enlarged it by means of a probe-pointed bistoury. He then extracted the exfoliation in three portions, which, when put together, fitted accurately.]

Polypus of the Nose.—This healthy-looking young woman complains that she cannot breathe through either nostril, and on looking into the right one I see a substance which presents the characters of what is called the simple or mucous polypus. As the nose is small, and the nostrils are very narrow, it might be supposed that the growth thus within reach of observation is sufficient to account for the symptoms, but from the complete obstruction of respiration and also the sound of the patient's voice, I suspect that there is an extension into the throat. I do not see anything here on looking in, but when my finger is introduced behind the soft palate, I feel a round tumor protruding from the posterior opening of the nasal cavity.

In considering the means that should be employed for the removal of this polypus, I beg to remind you that non-malignant growths are very limited in regard to the seat of their origin from within the nose, as they never proceed from the floor, septum, or external wall, and always do so from the roof, or that part which is formed by the turbinated plates of the ethmoid bone. Further, I must remind you that each nostril affords very little space in its transverse direction, and that the narrow slit which constitutes it is divided longitudinally into two nearly equal portions by the inferior spongy bone. Now the mucous polypus, always originating from the small extent of surface that has been thus defined, whether it does so singly or, as more frequently happens, in groups, descends so as to occupy first the higher and then the lower channel of the nose, but cannot attain any greater size so long as it is limited to these situations; and not possessing sufficient vigor of expansion to enlarge the osseous space, in order to attain any considerable bulk, must therefore proceed backwards towards the pharynx. Keeping these things in mind, you will at once perceive that the plan of removing polypus by means of a thread of wire, whether simply put up the nose, or passed through it into the throat, cannot possibly extirpate the growth, or do more than remove a portion of its substance. You will also require no arguments to prove the inutility and inexpediency of trying to accomplish the object in view by scissors, knives, or caustic; so that our choice is thus limited to evulsion by forceps.

This operation is generally regarded as very unsatisfactory, from the relief which it affords being seldom complete, and still less frequently permanent. But these imperfect results should in a great measure be ascribed to the faulty nature of the procedure usually employed, which is, aiming at the body of the growth to be removed instead of its narrow root, so as to detach merely the most projecting portions, instead of extirpating the whole mass entire. It is therefore no wonder that the operator fails to relieve the patient, or finds many "sittings," as he calls them, necessary, and nibbles away, in vain attempts to effect a radical cure. The forceps that were formerly employed on such occasions are indeed of themselves sufficient to show the utter hopelessness of succeeding by their means, since the blades are so large that they could not be insinuated into the narrow channel where the root of the growth is attached. You should select for the purpose an instrument such as that in my hand, which is of the smallest possible size, consistent with the requisite degree of strength; introduce it gently along the upper passage of the nose, with the blades separated so far as the walls of the cavity permit, until its further progress is arrested by the neck of the polypus; then firmly compress the handles, and, by a combination of traction with torsion in one uniform direction, break through the connecting textures included. If the growth does not follow the instrument, it must be introduced again and again in the same way until the nostril is completely cleared, when a piece of lint may be introduced to prevent further bleeding.

[Mr. Syme then proceeded in the way that has been described, and after several introductions of the forceps, succeeded in removing a large round polypus quite entire, immediately after which the patient breathed with perfect freedom through both nostrils.]

It seems not unworthy of notice that in this case there are some points of resemblance to that remarkable tumor named fibrous polypus, of which the characters are unity of growth, ligamentous toughness of consistence, extreme firmness of attachment, and disposition to bleed. Several cases have occurred here in which the patients had nearly died of hæmorrhage. and all the strength that I could exert was hardly sufficient to effect extraction, while blood streamed in torrents from the nose and mouth. But as this disease is not malignant, the operation, however formidable, proves satisfactory in its result. Now, you may have remarked that the tumor which has just been removed was solitary, and confined to one nostril, instead of occurring in both, as the mucous polypus almost always does; also that its connections were very firm, and that the bleeding was considerable. It would seem as if there may be conditions intermediate between the two sorts of growth, in which the respective characters more or less closely approach those of each other. For instance, some years ago, a nobleman returned from public service in one of the colonies, on account of complete obstruction to breathing through his nose, with a constant discharge of watery fluid from it, and occasionally alarming hæmorrhage, of which the amount was estimated not by ounces but by pints. He remained in London for two months under the care of several practitioners, who regarded the disease as incurable; and finding himself thus given up as hopeless, he applied to me, bringing with him a letter from the late Mr. Barnsby Cooper, which stated that the result of repeated consultations with Sir B. Brodie, Mr. Travers, Mr. Cæsar Hawkins, and himself, was a decision against interfering with the disease, which they seemed to regard as a tumor of the throat. Seeing no sign of malignant disposition in the aspect of the patient, who was about thirty-four years of age, and recognising in the tumor the characters which were familiar to me as those of a nasal growth stretching backwards into the pharyx, I at once undertook to remove the source of complaint. The patient and his friends at a distance having given their consent, I introduced the forceps, and at the very first pull extracted the whole growth, with immediate, complete and permanent relief. Four years have now elapsed, and there has not been the slightest symptom of relapse to interfere with the enjoyment of perfect health, or the vigorous exertion of an active life.

---

Lecture ii. Stricture of the Urethra.—There are two patients about to leave the hospital, whose cases seem to afford strong evidence in favor of treating obstinate structures by external incision. One of them, a sea-faring man of thirty-five, had suffered from the disease for upwards of fifteen years, and been under the care of various practitioners without obtaining relief. He was advised to seek my assistance by a surgeon of Halifax, in Nova Scotia, as laboring under an impermeable stricture, and, with this view, crossed the Atlantic. He is now, you see, perfectly well, and proposes returning to America, in full enjoyment of the comfort to which he has been so long a stranger. The other case is that of a young man of twenty six from Banffshire, who says that from boyhood he has, more or less constantly, suffered from irritation connected with his urinary organs. Latterly the disease had assumed its most aggravated form, being attended with fistulous openings and great swelling of the perinæum, with a never-ceasing involuntary discharge of urine, whilst he labored under a total inability of expelling it except by drops. At the first attempt I succeeded in passing a small bougie, and, without loss of time in the trial of other less efficient measures, divided the stricture upon a grooved director in the ordinary way. This patient also, you see, desires to go home with his urethra perfectly free, and all the distressing symptoms he had so long endured completely removed. So far as the immediate effects of the operation are concerned, it is impossible for any evidence to be more satisfactory than that which is afforded by these two cases. But it may be asked, will the relief prove permanent? There has now been sufficient length of time for proving that, even in the most extreme degree of inveteracy, obstinacy, and aggravation, a lasting recovery may result from this mode of treatment, without the use of bougies or other means to prevent relapse; and if the disease has occasionally returned, such exceptions should therefore be attributed to some faulty procedure, or other specialty of the case, rather than be regarded as constituting an objection to the operation itself. It is now in my power, through the kindness of Dr. Watson, house-surgeon to my late lamented colleague, Dr Mackenzie, to show you how perfectly the most tightly contracted urethra may be restored to a perfect condition by the procedure in question.

J. R——, aged forty-four, a fisherman, from Prestonpans, was admitted into the hospital on the 17th of March, 1852, on account of a stricture in the urethra of sixteen years' standing. Previously to admission he had been treated by bougies on various occasions, and under this mode of treatment had always suffered severely from rigors and febrile symptoms, without experiencing any benefit of longer duration than the period of dilatation. Latterly his symptoms had become more aggravated, and every attempt to pass instruments had proved ineffectual. A full sized instrument was arrested at the bulb, and, not without considerable difficulty, a No. 1 bougie was introduced into the bladder. The introduction of this instrument was followed by a severe rigor, and next day the patient suffered from retention of urine, to relieve which a No. 1 catheter was introduced. Dr. Mackenzie made repeated attempts to continue the treatment by bougies, but the constitutional disturbance produced by them was so severe, that he determined without further delay to divide the stricture. Accordingly having introduced a grooved staff, the size of a No. 1 bougie, into the bladder, he made an incision in the middle line of the perinæum, over the induration at the seat of stricture, and divided the contracted portion of the urethra to the extent of about three-quarters of an inch. A No. 8 catheter was passed with great facility into the bladder, and retained there, not more than half an ounce of blood being lost either during or after the operation. There was subsequently a slight degree of febrile disturbance, which subsided when the catheter was removed, at the end of forty-eight hours. During the following ten days, the urine passed in nearly equal proportions by the urethra and the wound, but before the end of three weeks had entirely resumed the natural channel. He remained in the hospital a few weeks longer, during which a full-sized bougie was passed occasionally without the slightest difficulty, and was dismissed on the 12th of June. He was seen again on the 9th of June, 1853, when a No. 12 bougie was passed with ease; and the patient stated that, although he had been much exposed to cold and wet in deep-sea fishing, he had never experienced the slightest difficulty of micturition. In April, 1854, he applied for admission to the hospital on account of symptoms supposed to be rheumatic, but which were traced to aneurism of the innominata. He suffered from no urinary symptoms whatever, but was admitted on account of the aneurism, and died in the beginning of June.

The symphysis pubis, bladder, and urethra, are now before you, and the symphysis has been divided, so as to allow the urethra and neck of the bladder to be laid open from above. A white depressed line of cicatrix, on the inner surface of the urethra, extending in the mesial line, half an inch forward from the extremity of the bulb, corresponds exactly with the line of cicatrix in the integuments and intermediate textures. The urethra in this part is rather wider than natural, from having a slight funnel-shaped depression on the lower surface, and, before being opened, easily admitted a No. 12 bougie. In other respects the coats of the urethra present a perfectly natural aspect, and the spongy tissue of the bulb, as well as the neighboring parts, are free from induration. There are two false passages in the prostate, evidently of very old standing. They are both seated at the upper surface of the urethra, one of them commencing at the triangular ligament, and forming a cul-de-sac in the prostate; the other, confined to the gland. The bladder, with the exception of being a little thicker than natural, presents nothing remarkable.

No one, looking at the urethra, which has remained thus sound and ample at the part where it had been so long and so tightly contracted, although two years had elapsed since the operation, without any means being used to prevent relapse, can entertain a reasonable doubt as to the recovery proving permanent, however long the patient might have lived; and such a result should stimulate our exertions to discover, so that they may be avoided, the errors of performance to which any recurrence of the symptoms that may have happened should be attributed, rather than to the principle of the operation.

I may here read to you a letter which I have just received from a patient who was sent here four years ago, by Mr. Harvey, of Lincoln, in circumstances of the most hopeless and distressing kind;—

"Sir:—In reply to your letter, I have to inform you that I never had better health in my life than I enjoy at the present time, that the stricture is quite removed, and that I have never felt anything from it since I underwent the operation.

(Signed) J. D."

This letter is dated Sheffield, and addressed to Mr. Harvey, whom I had requested to ascertain the patient's present state.

But the parts now before you afford a no less instructive lesson in regard to another most important point in the treatment of stricture. This is, the position of the disease respecting which the most vague and conflicting opinions still exist in the profession, as you may judge from one of the most esteemed authorities, Sir B. Brodie, representing the membranous portion of the urethra as the most common seat of contraction, while I am prepared to prove that it never occurs in this part of the canal. The erroneous opinions which exist on the subject have proceeded from two sources. Of these, the first is the difficulty of discriminating between the resistance to passing bougies into the bladder, which is caused by the contracted part of the canal grasping the instrument that has been carried through it, from an obstruction to the progress of the point; while the second is the deceitful feeling of a false passage, which allows the introduction, without much force so far as the surrounding textures are soft and yielding, but cannot be extended beyond any firm texture, or still less permit an entrance into the bladder. The truth is, that if you divide the urethra into three portions, of prostatic, membraneous, and the remaining part, which is covered by the corpus spongiosum, it is to the last alone that all strictures may be referred. However completely any one might be satisfied in his own mind as to this, he would probably find it impossible to convey the same persuasion to others, so long as he merely appealed to the practicability of passing instruments through the strictures alleged to exist in the posterior part of the canal, because success in doing so might be attributed to chance or good fortune on the part of the operator. But having now had occasion to operate more than a hundred times upon strictures of every sort and degree, by cutting externally upon a grooved director, so that the precise seat and extent of the incision could be determined, and having never been prevented from passing a full-sized instrument into the bladder, although I have never cut beyond the bulbous part of the urethra, I am entitled to conclude, and without any qualification, to affirm, that the fact is as has just been stated. Now, if you look at the prostatic portion of this urethra, you will see that various false passages have been made into this substance, so as to establish a cul-de-sac, which must plainly have resulted from the pressure of an instrument; and there can be as little doubt, that when the operators found their progress obstructed here, they believed that they had to deal with an impermeable stricture at the neck of the bladder. But you see that Dr. Mackenzie did not cut further back than the bulb, that he was nevertheless able to pass, without difficulty, a full-sized catheter into the bladder, and that the patient was completely relieved from his symptoms. There is at present under my charge a gentleman who has come from England, having been from home nearly two years, under the treatment of an eminent practitioner, who, he says, introduced instruments, with few exceptions, every day, for the remedy of a stricture which, he said, was seated at the verumontanum. You will now be able to understand the explanation of such a case.

The next patient that I shall bring before you is Edward Q——, aged fifty-five, a recruiting serjeant in the East India Company's service, enjoying good health, with the exception of stricture of the urethra, which has probably existed for upwards of twelve years; he had retention of urine in 1843, and was at that time treated with bougies, and remained pretty free from annoying symptoms till February last, when he again had retention after fatigue and exposure to cold; a good deal of bleeding followed the introduction of a catheter by the surgeon to whom he applied for relief, and ever since micturition has been only in a very small stream or in drops, with more or less incontinence of urine. On his admission, six weeks ago, I opened a large abscess in his perinæum, and thereby rescued him from danger; for if the abscess had opened internally, before it had found vent at the surface, urine would have got in, with great risk of serious consequences; as it was, the matter evacuated was free from urinous odour, and no urine was discharged from the external aperture till some days after the abscess had been opened. I have since employed dilatation, apparently with good success, in so far as a bougie of a considerable size can now be passed with facility into the bladder, whereas at first only the smallest instrument could be introduced through the stricture. The patient, however, has derived no benefit as regards his symptoms; micturition is still laborious and frequent, and not much better than in drops, and the clothes are still constantly wet from incontinence of urine. Incontinence is a frequent, though by no means a constant symptom of stricture; and, when present, is always a sign of a troublesome case. I have just received a letter from his commanding officer inquiring when a medical board may be held upon him, as his situation requires an active and efficient person to fill it. It is clear, that although strong and healthy, except as regards his local complaint, yet if now brought before a medical board, he must be invalided. What would be our condition if bougies alone could be resorted to? It is said by some, that if bougie can be introduced, the patient can be cured by dilatation. I should like the gentlemen who say this to make trial of the present case. You might introduce bougies here for weeks, and I believe for years, without benefit. But I am happy to say, that we have an alternative to look to, by which we shall, I believe, relieve him—viz., division of the peculiar contractile texture by external incision. It has been said, that I have intended this operation to supersede dilatation; but this is far from being true, for in ordinary cases I use dilatation as a gentler means, and reserve external incision for cases of peculiar obstinacy. I have now under my care, a gentleman who has come upwards of 6000 miles, willing to suffer anything for relief from a stricture, of which I have now cured him, by dilatation. At the same time, I think it quite possible that external incision may hereafter be proved so simple and satisfactory, that patients may come to prefer it to dilatation.

This operation, when first proposed, met with violent opposition, but this is no more than was to be expected; for a new proposal of any value is sure to be strongly opposed, while useless and injurious ones are, curiously enough, often received with great favor. To compare small things with great, I may remind you of the strong opposition made to Harvey's theory, of the circulation of the blood, and again to vaccination. To take another instance that has fallen under my own observation, five and twenty years ago excision of the elbow joint had not yet been performed in Great Britain, and when I first adopted it the most vehement opposition was raised. The late Mr. Liston, in this theatre, characterized it as a blackguard operation, but it has since come to be generally adopted.

This operation for stricture has passed through the period of opposition, and is now exposed to the danger of being adopted by persons who certainly do not, and either cannot or will not, understand the principles upon which it is founded. These principles may be said to be:—

First.—Stricture is never seated posteriorily to the bulb, and therefore the incision should never extend further back than the bulb.

Second.—A grooved director must be insinuated through the contracted part, without injury or abrasion of the lining membrane of the urethra.

Third.—If the incision has been properly performed, there is no need to dilate sinuses, which are sure to close when the stricture is removed.

Fourth.—A catheter should be introduced into the bladder after the operation, and retained for forty-eight hours; not less, on account of the risk of extravasation of urine; and not longer, because it is unnecessary, and apt to do harm.

You may fancy with what astonishment I read in the journals from week to week, statements to the effect that strictures are very often seated in the membranous part of the urethra, and that incisions are therefore required there, or even at the neck of the bladder. I also find it stated that in cases of impermeable stricture the bougie must be pushed onwards by force in the direction which the anatomical knowledge of the surgeon leads him to suppose to be the course of the urethra. In a journal published the other day, a case is recorded, in which the operator is said to have divided a stricture upon a silver catheter. Consider for one moment the danger of such a proceeding; even if the urethra were healthy there would be great danger of wounding the artery of the bulb if the knife should glide off the catheter; but considering the induration of the parts divided, and the uncertainty of the precise seat of contraction, I do not hesitate to assert that it is impossible to divide a stricture with certainty or safety in this way. For my own part, I would as soon think of dancing on a tight rope as of making such an attempt. It is further reported that in this case a number of sinuses were laid open, and that a catheter was retained in the bladder for eleven days, and yet it is said by the reporter that this operation was conducted emphatically after my manner. I say emphatically that it was not conducted after my manner, but was a miserable travesty of it, which would be simply ludicrous were it not that its result was so tragical.

[The patient having been brought into the theatre, and chloroform administered, Mr. Syme passed a No. 2 bougie into the bladder, and afterwards introduced a No. 9 bougie for a short distance into the urethra, to make sure that a stricture near the orifice had been dilated sufficiently to admit the thick part of his new staff. This is a steel instrument of the same general form as a catheter of short curve, with the curved part of the size of a No. 1 or No. 2 bougie, and deeply grooved on the convexity. The rest of the instrument up to the handle is of the size of the No. 9 bougie, and destitute of a groove. The groove in the thin part terminates anteriorly in a notch in the abrupt commencement of the thick part. Having introduced this instrument through the stricture, he made an incision, above two inches long, in the raphe of the perinæum, and cut carefully down in the middle line, through the mass of indurated textures, till he felt the staff with the fore-finger of his left hand. Taking the staff into his own hand, he introduced the point of the knife into the groove, about an inch and a quarter behind the commencement of the thick part, holding it with the handle upwards, and the back supported in its entire length on the fore-finger, which he was thus able to use to feel where the knife was going, while it removed all risk of the point of the instrument slipping deeply on either side of the staff. Having depressed the handle, he then pushed the knife forward along the groove, through the tough substance of the stricture till the point was arrested by the notch at the commencement of the thick part of the staff. To ensure the complete division of the anterior part of the stricture, he now pushed forward the staff and knife together for a short distance. He then introduced into the bladder a No. 11 catheter, placing the left index finger in the wound as a guide. This was necessary on account of the great depth of the incision through the indurated perinæum, which measured full two inches. The catheter was provided with a stop-cock, and the end curved downwards to prevent the inconvenience of the trickling of urine upon the body. Every thing went on favorably, the urine being discharged from the wound entirely for a week, and then gradually resuming its natural course, so that a fortnight after the operation, a few drops only escaped by the wound, the swelling of the perinæum had entirely disappeared, he no longer suffered from incontinence, and passed his urine in a good stream. The operation was performed on the

27th of November, and the patient was discharged fit for duty on the 16th of December.]

---

LECTURE III. CHLOROFORM.—I have now to speak of some cases in which chloroform will be given as usual, in consequence of the pain otherwise attendant on the operations to be performed; but before the patients are brought in I may take this opportunity of saying a few words regarding the use of chloroform, as you see that fatal cases, I am sorry to say, still occur, and that the medical journals, consequently, express doubts as to the use of chloroform at all, or say that, if used, it must be only with the greatest caution. Chloroform is no doubt a very powerful agent, sufficient to destroy the strongest individual if employed freely enough. Its fatal effects were shown at an early period in the following way: a lecturer in London was illustrating its action upon a guinea pig, which was placed under a glass jar along with some of the anæsthetic; the professor, in the eagerness of his discourse, left the animal too long under the jar, and it died. He explained to his audience the cause of the accident, but though the reasons he gave were satisfactory, yet an impression was made upon the public which was not soon effaced. In this respect, however, there is nothing peculiar to chloroform as a medicinal agent; opium, prussic acid, strychnia, &c., not only may, but often do destroy life, through being used in overdoses. But the question is, may it be used, judiciously, so as to do the good without exposing the patient to the risk of the evil! It is said in London that it cannot; that the risk is so great that it is only justifiable to use it in case of operations accompanied with an extreme degree of pain, or where stillness on the part of the patient is essential to success, and that the greatest caution is required in its administration; here we say that, if used with moderate care, it is perfectly safe. It was in this theatre that chloroform was first administered in public, by Dr. Simpson, seven years ago; since then it has been almost daily given here, yet we have not had a fatal case. It is true one solitary instance of death from chloroform has occurred in another part of the hospital, but that case has nothing to do with us; so far as my department is concerned, it might as well have been at Guy's Hospital, or in Kamschatka, or anywhere else; indeed, it so happened, that at the very time when that unfortunate event was taking place in another part of the establishment, I was myself performing an operation on a patient under chloroform in this theatre.

In inquiring into the reason of this difference between the experience of chloroform in London and here, we have not far to search for the explanation: it must lie in one of three things—viz., difference in the chloroform, difference in the patients, or difference in the mode of administration: with regard to the chloroform, I believe that most of that which is used in London is made in Edinburg, and I know that some of the fatal cases might be shown to have occurred with Edinburg chloroform.

With respect to the patients, it appears that great care is taken in London to use chloroform only in persons free from chest affections, especially cardiac derangements: here we never ask any questions as to the state of the heart or constitution of the patients. In all cases where chloroform is required for an operation, it is freely given. Now, considering the frequency of cardiac disease, and particularly of fatty heart,—which in fact, is I believe rarely absent, at any rate in elderly persons,—and considering also the immense number of patients operated on, you cannot doubt that many hundreds with fatty degeneration of the heart have had chloroform administered to them here; we even give chloroform without scruple, where we know disease of the heart exists. Within the last week, a case in point occurred in my practice. A patient, with great dread of pain, and also with a horror of chloroform, had long endured severe pain from his disease, till existence had become a burden, because he could not venture to undergo the necessary operation without chloroform, while his medical adviser considered that to take it would be for him almost certain death, on account of organic disease known to exist in the heart; for which he had consulted, and which it may be remarked had been recognized by Dr. Addison, of Guy's Hospital. At length his medical attendant said to him, that he had suffered so much from his complaint, that even if he died under chloroform this would be better than remaining as he was; while, if it should so happen that he should recover, he would be able to enjoy the rest of his life free from the disease. The patient could not resist the force of this argument, and came to Edinburg prepared for either alternative. I performed the operation under chloroform; and the first thing that he did on waking was to ask for a cigar.

As another example, I may mention the case of an old gentleman, aged seventy-four, affected with disease of the heart, from whose bladder I removed a large stone, some years ago. Chloroform was administered as he lay in bed; and he was put so fully under its influence, that he was taken from the bed, was operated on, and put back to bed, before he awoke from his sleep. He recovered perfectly; but some time afterwards died, and Dr. Begbie, on examining the body, found the disease of the heart, which had been diagnosed during life. We cannot, therefore, attribute the absence of deaths here to our being more discriminating than others in the patients to whom we administer chloroform; the very reverse, in fact, being the case.

I think then, gentlemen, that we are necessarily led to the conclusion, that the difference of results depends on difference in the mode of administration. We know that in other cases differences in the method of procedure have led to differences of results—*e. g.*, it was said in London, that amputation at the ankle invariably caused sloughing; but it turned out that the surgeon who made this statement, performed the operation in a manner that deviated much from the principles on which it is here performed successfully, and which led inevitably to sloughing. So of dividing stricture by external incision, it was said that extravasation of urine must necessarily take place in a dangerous manner, and that other serious complications may occur; but it turned out that the incision had been made without guide on a silver catheter—in short, with such deviations from principles as fully to account for the results.

So far as I can ascertain, from what I have heard and read upon the subject, there are important differences between the mode of administration of chloroform here and in London. It appears that here it is given according to principle, there according to rule. There great attention is paid to the number of drachms or minims employed; here we are entirely regardless of the amount used, and are guided only by the symptoms of the patient. The points that we consider of the greatest importance in the administration of chloroform are—first, a free admixture of air with the vapour of the chloroform, to ensure which, a soft porous material, such as a folded towel or handkerchief, is employed, presenting a pretty large surface, instead of a small piece of lint, or any other apparatus held to the nose. Secondly, if this is attended to, the more rapidly the chloroform is given the better, till the effect is produced; and, hence, we do not stint the quantity of chloroform. Then—and this is a most important point—we are guided as to the effect, not by the circulation, but entirely by the respiration; you never see anybody here with his finger on the pulse while chloroform is given. So soon as breathing becomes stertorous we cease the administration; from what I have learned, it is sometimes pushed further elsewhere, but this we consider in the highest degree dangerous. Attention to the tongue is another point which we find of great consequence. When respiration becomes difficult, or ceases, we open the mouth, seize the tip of the tongue with artery-forceps, and pull it well forward; and there can be little doubt that death would have occurred in some cases if it had not been for the use of this expedient. We also always give the chloroform in the horizontal position, and take care that there is no article of clothing constricting the neck. There are thus considerable differences between our practice and that which prevails more or less elsewhere. We use no aparatus whatever, take the respiration for our guide, attend to the condition of the tongue, and never continue beyond the point when the patient is fully under the influence of the anæsthetic.

You observe that in this matter I am very far from taking any credit to myself; all that I have done has been to follow the example of Dr. Simpson, and all that I would say respecting our brethren in London, is, that they have not been so fortunate as to get into the right way in the first instance, and I would urge upon them to banish all previous notions, and to keep in view the essential points to which I have alluded; then, if unfortunately there should still be fatal cases, I shall not presume to speak further upon the subject. As the matter at present stands, the discussions prevalent in the profession tend to give the public a dread of chloroform, and to limit the advantages which it confers; and so long as the difference of opinion seemed due to important difference of practice, I felt called upon to address to you the observations I have made.

---

FISTULA IN ANO.—The next case is one requiring a little operation, one of fistula in ano. Fistula in ano is so frequent in this hospital as to make it necessary for me to warn gentlemen from time to time against supposing it endemic in Edinburgh, the truth being that the greater number of the patients come from the country, many of them from the very remotest parts. This may surprise you, when you consider that the complaint is of very small extent, and rather inconvenient than serious, and that the operation for its relief is of the very simplest kind; and in order to explain the matter, it will be necessary to go into the history of the subject, which may be instructive, as showing how long and how difficult a process is the diffusion of an improvement in surgery. It was known from time immemorial that abscess frequently occurred near the verge of the anus, and that the cavity was apt to be only partially obliterated, leaving a sinus, which was called fistula in ano, the obstinacy of which was supposed to be due to a peculiar action of the part; and accordingly it was treated very energetically, being cut out, or subjected to the action of caustics, or sometimes cut into, and then cauterized. We are indebted to the distinguished member of the profession to whom I alluded on a recent occasion,—viz., to Percival Pott,—for showing that the obstinacy of the fistula was due to peculiarity of position with respect to surrounding parts, and not to anything specific in the surface. He pointed out that a partition existed between the fistula and the rectum, and that all that was necessary was to throw the two cavities into one by dividing the partition. The commanding position occupied by Pott, as senior surgeon to the largest hospital in London, gave great influence to his statements, and the practices of excision and cauterization were consequently soon exploded in this country; but in some other places the old system is still employed. I have myself seen a fistula cut out; and if you go abroad to see the foreign "improvements" in practice, you will very likely somewhere or other see fistula cut out or cut into. But Pott's operation, although a great improvement on the former practice, was still by no means satisfactory. He supposed that it was necessary to cut to the top of the sinus, and, as this often ran high up, the operation was accompanied with great pain, and with an amount of hæmorrhage sometimes alarming, or, as it has been said, even fatal; and unless the edges of the wound were kept open by daily dressing, union was apt to occur externally, while a part of the cavity continued unobliterated; and thus the operation often failed to give the desired relief, so that it was regarded as a painful one, requiring long confinement to bed, and in the end uncertain of its effect.

About thirty-five years ago, a French surgeon, M. Ribes, called the attention of the profession to an important point in the pathology of fistula; for while surgeons commonly regarded it as of three characters—viz., blind external, when it opened only at the surface; blind internal, when it communicated with the rectum, but had no opening externally; and complete, when both an external and an internal opening were present—he affirmed that both openings always existed, and that the idea of blind external fistula had proceeded from an error of observation respecting the position of the internal aperture, which had been always sought for at the top of the sinus, whereas he showed it to be placed within one inch, or at most an inch and a quarter, from the orifice of the rectum, however high the sinus might extend; and he also pointed out, that provided the incision included the internal orifice, it was sufficient for the cure of the disease. Here you see an improvement pathological and practical. The operation caused very little bleeding, no subsequent dressing was required, and the effect was certain.

It happened that my friend and colleague, Dr. Christison, being in Paris in 1821, became acquainted with the observations of M. Ribes, and on his return here mentioned them to me, at that time house-surgeon to this hospital, and directed me to the *Archives générales*, in which they were published. I took every opportunity of testing the truth of this new and startling statement, and found it to be substantially correct. On referring to M. Ribes' paper, however, the explanation which he gave of the origin of fistula appeared to me unsatisfactory. He supposed that it always began by ulceration of the mucous membrane of the rectum, after which portion of the contents of the bowel escaped into the surrounding textures, and gave origin to abscess there; but I noticed that on opening the abscess no internal aperture was to be discovered by the most careful examination, and that the matter evacuated was not mixed with fæculent or gaseous material, but simply a small quantity of well-digested pus. I also observed that a fistula of seven days' (or even weeks') standing, had, generally, no internal opening, and it therefore appeared to me, that the mucous membrane of the rectum, although thinned and denuded by the abscess, did not give way until after the matter had found vent at the surface, and the external orifice had closed to some extent, so as to confine the pus, and thus cause ulcerative absorption. This, however, is a matter of curiosity, rather than of practical utility. Notwithstanding the importance of the facts observed by M. Ribes, and the publicity which he gave to them, and notwithstanding the efforts which have been ever since made here by myself, and, for ought I know, by others elsewhere, to extend the knowledge of them, yet the greater number of surgeons for a long time obstinately refused to admit their truth, and to modify their practice accordingly.

In 1836, about sixteen years after the publication of M. Ribes' paper, Sir B. Brodie wrote thus:—"If the internal opening be at the upper extremity of the sinus, the operation is simple enough. You indroduce the forefinger of one hand into the rectum, and with the other hand you direct the curved, probe-pointed bistoury through the external opening into the sinus, and afterwards through the internal opening into the rectum; then, keeping the probe point in contact with the forefinger, you draw the instrument downwards, dividing all the parts below it. If the internal opening be anywhere in the middle part of the sinus, you proceed in the same manner, but a second incision is then necessary, to lay open the upper extremity of the sinus. The probe point of the bistoury must be made to penetrate the tunics of the rectum before this second incision is made. If the sinus has no communication with the rectum, the tunics of the latter must be penetrated as near as possible to the upper extremity of the sinus, the incision being made afterwards in the manner which has been just explained."

In 1837, I published a treatise on "Diseases of the Rectum," in which I explained very fully the views of M. Ribes, and also pointed out the error into which I believed he had fallen with regard to the origin of the disease. In 1844, Sir B. Brodie writes: —"The first thing to be done is to find the inner opening. I do not say that you will always succeed in finding it—certainly not the first time; but you will rarely fail if you look for it in the right place. Formerly I often failed, and for this reason—I did not know where to look for it. I used to think that it was to be found in the upper part of the sinus, but it is never found there if the sinus runs high up. You must search for it immediately above the sphincter muscle." Sir Benjamin does not say what his authority for this statement is, so we must suppose it to be original; but, if so, it is curious that, whilst discovering the truth made out by M. Ribes, he has also fallen into his error of supposing that the disease always begins by ulceration of the mucous membrane. For he say:—"I believe that this is the way in which fistulæ in ano are always formed—namely, the disease is originally an ulcer of the mucous membrane of the bowel, extending through the muscular tunic into the cellular membrane external to the intestine, and I will state my reason for entertaining that opinion. The matter is one of great interest as a question of pathology, but it is one of great importance, as I shall show by-and-bye, in connection with surgical practice. It is admitted by every one that in the great number of cases of fistulæ in ano there is an inner opening to the gut, as well as the outer opening; and I am satisfied that the inner opening always exists, because I scarcely ever fail to find it now that I look for it in the proper place, and seek it carefully. I have, in a dead body, examined the parts where fistulæ had existed several times, and in every instance I have found an inner opening to it. This affords a very reasonable explanation of the formation of these abscesses; it is almost impossible to understand on any other ground why suppuration should take place in the vicinity of the rectum, more than in any other part of the body, and why the cellular membrane there should suppurate more that cellular membrane elsewhere. Moreover, the pus contained in an abscess near the rectum scarcely ever presents the appearance of laudable pus; it is always dirty-colored and offensive to the smell—sometimes highly offensive, and occasionally you find feculant matter in it quite distinct." Now this I deny, and appeal to the abscesses which you may have seen, or will see before long, and also to the distinct statements of the patients themselves.

The discovery of the uniform existence of an internal opening near the anus may be said to have perfected the operation, but in consequence of the old errors having been so long prevalent, the treatment has not hitherto been nearly so satisfactory as it should have been in the profession at large; and hence the explanation of the fact, that patients effected with fistula in ano, come here from all parts of the county, under the impression that the operation they are about to undergo is a very serious one, and involves long confinement to bed. The operation, though very simple in principle, and easy of performance, is still one that requires care and patience. Whenever you examine a fistula of six weeks' or two months' standing, you must proceed on the supposition that an internal opening exists. The track that leads to it may be tortuous, but you must search carefully again and again, if you fail to find the aperture in the first instance, and be very slow to be persuaded that it is not there. A piece of lint is placed in the wound at the time of the operation, and the only other dressing required is washing the part occasionally with soap-and-water for a few days.

The patient R. M'L——, aged twenty three, a coach builder from Stirling, was then brought into the theatre, and stated that the disease had commenced as a small suppurating point near the anus, which caused no great pain, and broke off itself, discharging considerably less than a teaspoonful of pus, unmixed with feculant or gaseous matter. The external opening was very small and inconspicuous, but the internal opening was readily found, a little above the anus, by Mr. Syme, who, in order to demonstrate the existence of the inner aperture, and the small amount of soft parts between it and the surface, brought out the end of the probe at the anus, and cut out the probe with the bistoury, remarking that though he had pursued this course in the present instance for the sake of demonstration, yet it was one to be recommended in all cases where much difficulty was experienced in discovering the internal opening, as the most certain means of securing the object in view. A piece of lint was placed in the wound; no hæmorrhage whatever occurred after the few drops of blood that were lost during the operation. He was discharged on the 25th of December.—*London Lancet.*

